# Supplementary material for: Regulation of neuronal axon specification by glia-neuron gap junctions in C. elegans
Source: eLife. 2016 Oct 21;5:e19510. doi: 10.7554/eLife.19510 (PMC5083064; doi:10.7554/eLife.19510)
Supplement: Supplementay file 1. — DOI: http://dx.doi.org/10.7554/eLife.19510.013 [file elife-19510-fig1.docx]

Supplemental Table 1, Strain list

| Strain | genotype | Plasmid | Related Figures |
| --- | --- | --- | --- |
| CZ15726 | *Punc-25::gfp(juIs76) II; unc-30(ju32) IV* |  | Fig.1B |
| CZ15720 | *unc-30(ju32) Punc-25::snb-1::gfp(juIs1) IV* |  | Fig.1C D and control of SNB-1::GFP marker in all other figures |
| CZ15490 | *unc-30(ju32) Punc-25::snb-1::gfp(juIs1) IV; unc-1(ju1057) X* |  | Fig.1C D and Fig. 2A B |
| NYL55 | *unc-30(ju32) Punc-25::snb-1::gfp(juIs1) IV; unc-1(ju1057) X; Pdpy-30::unc-1,Pttx-3::rfp(yadEx7)* | PNYL165(50 ng/µl) | Fig. 1C D and 2A |
| NYL83 | *unc-104(rh43) II; unc-30(ju32) Punc-25::snb-1::gfp(juIs1) IV; unc-1(ju1057) X* |  | Fig. 1C D |
| NYL652 | *unc-104(rh43) II; unc-30(ju32) Punc-25::snb-1::gfp(juIs1) IV* |  | Fig. 1D |
| NYL103 | *unc-30(ju32) Punc-25::snb-1::gfp(juIs1) IV; unc-1(e580) X* |  | Fig.1D |
| CZ15614 | *unc-30(ju32) Punc-25::snb-1::gfp(juIs1) IV; unc-1(e2522) X* |  | Fig. 1D |
| CZ15725 | *unc-30(ok613) IV; unc-1(ju1057) X; Punc-25::syd-2::gfp(hpIs3)* |  | Fig. 1E and F |
| CZ15724 | *unc-30(ok613) IV; Punc-25::syd-2::gfp(hpIs3)* |  | Fig. 1E and F |
| NYL640 | *unc-30(ok613) IV; unc-1(ju1057) X; Punc-25::syd-2::gfp(hpIs2); Punc-25::rab-3::mcherry (yadEX288)* | PNYL163(20 ng/µl) | Fig. 1G |
| NYL22 | *unc-30(ju32) Punc-25::snb-1::gfp(juIs1) IV; unc-1(hs1ts) X* |  | Fig.1H |
| NYL398 | *unc-30(ju32) Punc-25::snb-1::gfp(juIs1) IV; unc-1(ju1057) X; Punc-25::unc-1,Pttx-3::rfp (yadEx164)* | PNYL78(50 ng/µl) | Fig.2A |
| NYL203 | *unc-30(ju32) Punc-25::snb-1::gfp(juIs1) IV; unc-1(ju1057) X; Pgly-18::unc-1, Pttx-3::rfp (yadEx76)* | PNYL75(50 ng/µl) | Fig.2A |
| NYL142 | *unc-30(ju32) Punc-25::snb-1::gfp(juIs1) IV; unc-1(ju1057) X; Punc-25::unc-1, Pgly-18::unc-1, Pttx-3::rfp(yadEX48)* | PNYL78(50 ng/µl) & PNYL75(50 ng/µl) | Fig.2A |
| NYL57 | *unc-30(ju32) IV Punc-25::snb-1::gfp(juIs1) IV ; unc-1(ju1057) X ; Punc-33::unc-1, Pttx-3::rfp (yadEx9)* |  | Fig. 2A |
| NYL1309 | *unc-30(ju32) IV Punc-25::snb-1::gfp(juIs1) IV ; unc-1(ju1057) X ; Pnep-2::unc-1, Pttx-3::rfp (yadEx635)* | PNYL357(50 ng/µl) | Fig.2A |
| NYL1311 | *unc-30(ju32) IV Punc-25::snb-1::gfp(juIs1) IV ; unc-1(ju1057) X ; Pmyo-3::unc-1, Pttx-3::rfp (yadEx637)* | PNYL609(50 ng/µl) | Fig. 2A |
| NYL1315 | *unc-30(ju32) IV Punc-25::snb-1::gfp(juIs1) IV ; unc-1(ju1057) X ; Pmyo-3::unc-1, Punc-25::unc-1, Pttx-3::rfp(yadEx641)* | PNYL609(50 ng/µl) and PNYL78 (50 ng/µl) | Fig.2A |
| NYL1313 | *unc-30(ju32) IV Punc-25::snb-1::gfp(juIs1) IV ; unc-1(ju1057) X ; Pmyo-3::unc-1, Pgly-18::unc-1, Pttx-3::rfp(yadEx639)* | PNYL609(50 ng/µl) and PNYL75 (50 ng/µl) | Fig. 2A |
| NYL1083 | *unc-30(ju32) IV Punc-25::snb-1::gfp(juIs1) IV ; unc-1(ju1057) X ; Punc-25::unc-1, Pnep-2::unc-1, Pttx-3::rfp(yadEx534)* | PNYL357(50 ng/µl) and and PNYL78 (50 ng/µl) | Fig. 2A |
| NYL1404 | *unc-30(ju32) Punc-25::snb-1::gfp(juIs1) IV; unc-1(ju1057) X; Pegl-6::unc-1, Pttx-3::rfp (yadEx669)* | PNYL550(50 ng/µl) | Fig. 2A |
| NYL1410 | *unc-30(ju32) Punc-25::snb-1::gfp(juIs1) IV; unc-1(ju1057) X; Pegl-6::unc-1, Punc-25::unc-1, Pttx-3::rfp(yadEx675)* | PNYL550(50 ng/µl) and PNYL78 (50 ng/µl) | Fig. 2A |
| NYL1408 | *unc-30(ju32) Punc-25::snb-1::gfp(juIs1) IV; unc-1(ju1057) X; Plet-2::unc-1, Pttx-3::rfp (yadEx673)* | PNYL589(50 ng/µl) | Fig. 2A |
| NYL1414 | *unc-30(ju32) Punc-25::snb-1::gfp(juIs1) IV; unc-1(ju1057) X; Plet-2::unc-1, Punc-25::unc-1, Pttx-3::rfp(yadEx679)* | PNYL589(50 ng/µl) and PNYL78 (50 ng/µl) | Fig. 2A |
| NYL1317 | *unc-30(ju32) IV Punc-25::SNB-1::gfp(juIs1) IV ; unc-1(ju1057) X ; Psur-5::mCherry::sur-5;Punc-25::mCherry;Pgly-18::mCherry;Pdpy-30::unc-1 (yadEx643)* |  | Fig. 2B |
| CZ15615 | *unc-30(ju32) Punc-25::snb-1::gfp(juIs1) IV; unc-7(e5) unc-9 (fc16) X* |  | Fig. 2C and 2D |
| CZ15633 | *unc-30(ju32) Punc-25::snb-1::gfp(juIs1) IV; unc-7(e5) X* |  | Fig. 2D E and F |
| CZ15634 | *unc-30(ju32) Punc-25::snb-1::gfp(juIs1) IV; unc-9(e101) X* |  | Fig. 2D |
| NYL197 | *unc-30(ju32) Punc-25::snb-1::gfp(juIs1) IV; unc-7(e5) X; Punc-25::unc-7, Pttx-3::rfp (yadEx70)* | PNYL64 (50 ng/µl) | Fig. 2E |
| NYL199 | *unc-30(ju32) Punc-25::snb-1::gfp(juIs1) IV; unc-7(e5) X; Pgly-18::unc-7, Pttx-3::rfp (yadEx72)* | PNYL76 (50 ng/µl) | Fig. 2E |
| NYL201 | *unc-30(ju32) Punc-25::snb-1::gfp(juIs1) IV; unc-7(e5) X; Punc-25::unc-7, Pgly-18::unc-7, Pttx-3::rfp(yadEx74)* | PNYL64 (50 ng/µl) & PNYL76 (50 ng/µl) | Fig. 2E |
| NYL196 | *unc-30(ju32) Punc-25::snb-1::gfp(juIs1) IV; unc-7(e5) X; Pdpy-30::cys-less unc-7, Pttx-3::rfp(yadEx69)* | PNYL81 (50 ng/µl) | Fig. 2F |
| NYL138 | *unc-30(ju32) Punc-25::snb-1::gfp(juIs1) IV; unc-7(e5) X; Pdpy-30::unc-7,Pttx-3::rfp (yadEx44)* | PNYL166(50 ng/µl) | Fig. 2F |
| NYL644 | *unc-30(ok613) IV; unc-1(e580) X; Punc-25::gfp::ebp-2, Pttx-3::rfp(yadIs17)* |  | Fig. 3A and B |
| NYL210 | *unc-30(ok613) IV; unc-7(e5) X unc-9(fc16unc-9) X; Punc-25::ebp-2::gfp, Pttx-3::rfp (yadIs17)* |  | Fig. 3A and B |
| NYL581 | *unc-30(ok613) IV; Punc-25::gfp::ebp-2, Pttx-3::rfp (yadIs17)* |  | Fig. 3A and B |
| NYL671 | *cdk-5(ok626) III; unc-30(ok613 ) IV; Punc-25::gfp::ebp-2, Pttx-3::rfp(yadIs17)* |  | Fig. 4A |
| NYL305 | *cdka-1(tm0648) III; unc-30(ju32) Punc-25::snb-1::gfp(juIs1) IV* |  | Fig. 4B C and D |
| NYL207 | *cdk-5(ok626) III; unc-30(ju32) Punc-25::snb-1::gfp(juIs1) IV* |  | Fig. 4B C and D |
| NYL923 | *cdk-5(ok626) III; unc-30(ju32) Punc-25::snb-1::gfp(juIs1) IV; Punc-25::CDK-5, Pttx-3::rfp (yadEx168)* | PNYL293  (40 ng/µl) | Fig.4 C |
| NYL143 | *cdka-1(tm0648) III; unc-30(ju32 Punc-25::snb-1::gfp(juIs1) IV; unc-7(e5) X* |  | Fig. 4D |
| NYL144 | *cdk-5(ok626) III; unc-30(ju32 Punc-25::snb-1::gfp(juIs1) IV; unc-7(e5) X* |  | Fig. 4D |
| NYL720 | *unc-7(e5) X unc-9(fc16unc-9) X ; Punc-25::flag::cdka-1, Pttx-3::rfp(yadIs13)* |  | Fig. 4E and F |
| NYL387 | *Punc-25::flag::cdka-1, Pttx-3::rfp (yadIs13)* |  | Fig. 4E F and 5A B |
| NYL454 | *unc-1(e580) X; Punc-25::flag::cdka-1, Pttx-3::rfp (yadIs13)* |  | Fig. 4E F and 5A |
| NYL639 | *unc-30(ju32) Punc-25::snb-1::gfp(juIs1) IV; unc-1(ju1057) X; Punc-25::P35, Pttx-3::rfp (yadEX287)* | PNYL161(150 ng/µl) | Fig.4G |
| NYL636 | *unc-30(ju32) Punc-25::snb-1::gfp(juIs1) IV; unc-1(ju1057) X; Punc-25::P25, Pttx-3::rfp (yadEX284)* | PNYL160(150 ng/µl) | Fig.4G |
| NYL355 | *unc-30(ju32) Punc-25::snb-1::gfp(juIs1) IV; unc-1(ju1057) X; Punc-25::P25, Pttx-3::rfp (yadEX142)* | PNYL160(50 ng/µl) | Fig. 4G |
| NYL420 | *unc-30(ju32) Punc-25::snb-1::gfp(juIs1) IV; Punc-25::P25, Pttx-3::rfp(yadEX165)* | PNYL160(50 ng/µl) | Fig.4G |
| NYL1420 | *unc-30(ju32) Punc-25::snb-1::gfp(juIs1) IV; Punc-25::calb, Pttx-3::rfp(yadEx685)* | PNYL242(150 ng/µl) | FIG. 5D |
| NYL1416 | *unc-30(ju32) Punc-25::snb-1::gfp(juIs1) IV; Pgly-18::calb, Pttx-3::rfp(yadEx681)* | PNYL243(150 ng/µl) | FIG. 5D, 5F and 6B |
| NYL1418 | *unc-30(ju32) Punc-25::snb-1::gfp(juIs1) IV; Pegl-6::calb, Pttx-3::rfp(yadEx683)* | PNYL559(150 ng/µl) | FIG. 5D |
| NYL1422 | *unc-30(ju32) Punc-25::snb-1::gfp(juIs1) IV; Punc-25::calb Pgly-18::calb, Pttx-3::rfp (yadEx687)* | PNYL242(150 ng/µl) and PNYL243(150 ng/µl) | FIG. 5D |
| NYL1422 | *unc-30(ju32) Punc-25::snb-1::gfp(juIs1) IV; Punc-25::calb Pegl-6::calb, Pttx-3::rfp (yadEx689)* | PNYL242(150 ng/µl) and PNYL559(150 ng/µl) | FIG. 5D |
| NYL1451 | *unc-30(ju32) Punc-25::snb-1::gfp(juIs1) IV; Pmyo-3::calb, Pttx-3::rfp(yadEx714)* | PNYL537(150 ng/µl) | FIG. 5D |
| NYL1499 | *unc-30(ok613) IV; Punc-25::gfp::ebp-2, Pttx-3::rfp(yadIs17); Pgly-18::calb, Pttx-3::rfp(yadEx681)* |  | FIG. 5E |
| NYL1496 | *cdk-5(ok626) III; unc-30(ju32) Punc-25::snb-1::gfp(juIs1) IV; Pgly-18::calb, Pttx-3::rfp (yadEx681)* |  | FIG. 5F |
| NYL1498 | *unc-30(ju32) Punc-25::snb-1::gfp(juIs1) IV; Punc-25::P25, Pttx-3::gfp(yadEx753)* |  | FIG. 5F |
| NYL1497 | *unc-30(ju32) Punc-25::snb-1::gfp(juIs1) IV; Pgly-18::calb, Pttx-3::rfp(yadEx681); Punc-25::P25, Pttx-3::gfp(yadEx753)* | PNYL160(150 ng/µl) | FIG. 5F |
| NYL670 | *clp-4(ok2808) III; unc-30(ju32 Punc-25::snb-1::gfp(juIs1) IV; unc-7(e5) X* |  | Fig. 5H |
| NYL923 | *clp-4(ok2808) III; unc-30(ju32) Punc-25::snb-1::gfp(juIs1) IV; Punc-25::clp-4, Pttx-3::rfp (yadEX168)* | PNYL164(40 ng/µl) | Fig. 5G |
| NYL310 | *clp-4(ok2808) III; unc-30(ju32) Punc-25::snb-1::gfp(juIs1) IV* |  | Fig. 5G and H |
| NYL67 | *unc-30(ju32) IV Punc-25::snb-1::gfp(juIs1) IV ; slo-1(eg142lf) V* |  | Fig. 6A |
| NYL425 | *unc-30(ju32) IV Punc-25::snb-1::gfp(juIs1) IV ; slo-1(eg142lf) V ; unc-1(e580null) X* |  | Fig. 6A |
| NYL1498 | *unc-30(ju32) Punc-25::snb-1::gfp(juIs1) IV; slo-1(eg142) V; Pgly-18::calb, Pttx-3::rfp (yadEx681)* |  | Fig. 6B |
| NYL926 | *unc-30(ok613) IV ; slo-1(eg142lf) V ; Punc-25::EBP-2::gfp, Pttx-3::rfp(yadIs17)* |  | Fig. 6C |
| NYL927 | *unc-30(ok613) IV ; slo-1(eg142lf) V ; unc-1(e580null) X ; Punc-25::EBP-2::gfp, Pttx-3::rfp(yadIs17)* |  | Fig. 6C |
| NYL928 | *slo-1(eg142lf) V ; Prgef-1::flag::cdka-1, Pttx-3::rfp (yadIs13)* |  | Fig. 6D E |
| NYL929 | *slo-1(eg142lf) V ; Prgef-1::flag::cdka-1, Pttx-3::rfp(yadIs13); unc-1(e580null) X* |  | Fig. 6D E |
| CZ333 | *Punc-25::snb-1::gfp(juIs1) IV* |  | Fig1. S2A |
| NYL623 | *Punc-25::snb-1::gfp(juIs1) IV ; unc-1(e580null) X* |  | Fig1. S2A |
| NYL4 | *Punc-25::gfp(juIs76) II ; Pgly-18-mCherry(yadIs2) IV* |  | Fig2. S1A |
| NYL1000 | *Pgly-18::gfp;Pnep-2::mcherry(yadEx474)* | PNYL329(40 ng/µl) and PNYL278(40 ng/µl) | Fig2. S2B |
| NYL1398 | *Pegl-6::gfp Pnep-2::mcherry(yadEx663)* | PNYL551(40 ng/µl) and PNYL278(40 ng/µl) | Fig2. S2B |
| NYL1402 | *Plet-2::gfp Pnep-2::mcherry(yadEx667)* | PNYL561(40 ng/µl) and PNYL278(40 ng/µl) | Fig2. S2B |
| NYL1211 | *unc-30(ok613) IV; Punc-25::gfp::EMBT(yadIs20)* |  | Fig. S3 A and B |
| NYL1330 | *cdka-1(tm0648) III; Punc-25::snb-1::gfp(juIs1) IV ; unc-1(e580null) X* |  | Fig. S3 C |
| NYL1331 | *cdk-5(ok626) III; Punc-25::snb-1::gfp(juIs1) IV ; unc-1(e580null) X* |  | Fig. S3 C |
| NYL1332 | *clp-4(ok2808) III; Punc-25::snb-1::gfp(juIs1) IV ; unc-1(e580null) X* |  | Fig. S3 C |
