# Supplementary material for: Regulation of neuronal axon specification by glia-neuron gap junctions in C. elegans
Source: eLife. 2016 Oct 21;5:e19510. doi: 10.7554/eLife.19510 (PMC5083064; doi:10.7554/eLife.19510)
Supplement: Supplementay file 2. — DOI: http://dx.doi.org/10.7554/eLife.19510.014 [file elife-19510-fig2.docx]

Supplemental Table 2, Plasmid list

| plasmid | Description |
| --- | --- |
| PNYL77 | *Punc-25::gfp::unc-1* |
| PNYL67 | *Punc-25::gfp::unc-7* |
| PNYL65 | *Punc-25::gfp::unc-9* |
| PNYL165 | *Pdpy-30::unc-1* |
| PNYL75 | *Pgly-18::unc-1* |
| PNYL609 | *Pmyo-3::unc-1* |
| PNYL76 | *Pgly-18::unc-7* |
| PNYL78 | *Punc-25::unc-1* |
| PNYL357 | *Pnep-2::unc-1* |
| PNYL64 | *Punc-25::unc-7* |
| PNYL166 | *Pdpy-30::unc-7* |
| PNY81 | *Pdpy-30::cys-less unc-7* |
| PNYL159 | *Prgef-1::flag-1::cdka-1* |
| PNYL160 | *Punc-25::P25* |
| PNYL161 | *Punc-25::P35* |
| PNYL162 | *Punc-25::gfp::ebp-2* |
| PNYL163 | *Punc-25::rab-3::mcherry* |
| PNYL329 | *Pgly-18::gfp* |
| PNYL278 | *Pnep-2::mcherry* |
| PNYL164 | *Punc-25::clp-4* |
| PNYL293 | *Punc-25::cdk-5* |
| PNYL242 | *Punc-25::calb* |
| PNYL243 | *Pgly-18::calb* |
| PNYL559 | *Pegl-6::calb* |
| PNYL537 | *Pmyo-3::calb* |
| PNYL550 | *Pegl-6::unc-1* |
| PNYL589 | *Plet-2::unc-1* |
| PNYL551 | *Pegl-6::gfp* |
| PNYL561 | *Plet-2::gfp* |
| PCZ805 | *Psur-5::sur-5::mCherry* |
